# Cyanobacterial Septal Junctions: Properties and Regulation

**DOI:** 10.3390/life9010001

**Published:** 2018-12-20

**Authors:** Enrique Flores, Mercedes Nieves-Morión, Conrad W. Mullineaux

**Affiliations:** 1 Instituto de Bioquímica Vegetal y Fotosíntesis, Consejo Superior de Investigaciones Científicas and Universidad de Sevilla, Américo Vespucio 49, 41092 Seville, Spain; mercedes.nieves@su.se; 2 School of Biological and Chemical Sciences, Queen Mary University of London, Mile End Road, London E1 4NS, UK; c.mullineaux@qmul.ac.uk

**Keywords:** *Anabaena*, bacterial multicellularity, intercellular communication

## Abstract

Heterocyst-forming cyanobacteria are multicellular organisms that grow as chains of cells (filaments or trichomes) in which the cells exchange regulators and nutrients. In this article, we review the morphological, physiological and genetic data that have led to our current understanding of intercellular communication in these organisms. Intercellular molecular exchange appears to take place by simple diffusion through proteinaceous structures, known as septal junctions, which connect the adjacent cells in the filament and traverse the septal peptidoglycan through perforations known as nanopores. Proteins that are necessary to produce, and that may be components of, the septal junctions―SepJ, FraC and FraD―have been identified in the heterocyst-forming cyanobacterium *Anabaena* sp. strain PCC 7120 model. Additionally, several proteins that are necessary to produce a normal number of nanopores and functional septal junctions have been identified, including AmiC-type amidases, peptidoglycan-binding proteins and some membrane transporters. Available reports and reevaluation of intercellular molecular transfer data for some mutants of *Anabaena* suggest that the septal junctions can be regulated, likely by a mechanism of gating.

## 1. Introduction

**Figure 1:**
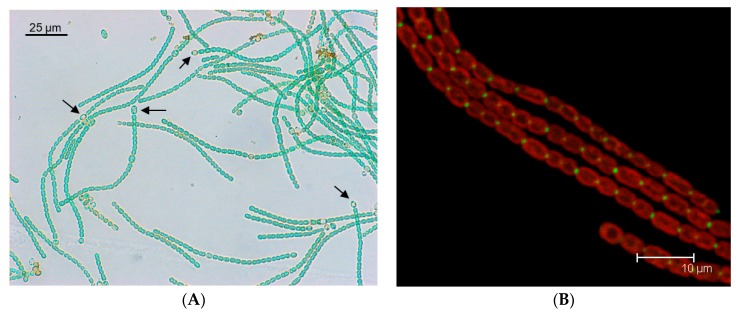
*Anabaena* sp. strain PCC 7120 and subcellular localization of the septal protein SepJ. (**A**) Bright field micrograph of filaments of *Anabaena* grown in BG11_0_ medium (lacking any source of combined nitrogen). Note the presence of heterocysts (some indicated by arrows). (**B**) Overlay of cyanobacterial autofluorescence (red) and GFP fluorescence (green) of *Anabaena* sp. strain CSAM137, which is PCC 7120 producing a SepJ-GFP fusion protein (see Reference [16] for details). Note the GFP foci at the intercellular septa, which show the subcellular localization of SepJ.

Heterocyst-forming cyanobacteria form chains of cells (filaments or trichomes) whose growth under conditions of deprivation of combined nitrogen depends on the activity of two cell types: vegetative cells that fix CO_2_ performing oxygenic photosynthesis, and heterocysts devoted to N_2_ fixation. The heterocysts are terminally differentiated cells that, in mature diazotrophic filaments, represent about 10% of the cells (Figure 1A). Heterocysts are formed by differentiation of vegetative cells, which takes place in response to nitrogen deprivation or, in diazotrophic filaments, when the distance between two heterocysts increases as the result of vegetative cell growth. Differentiation requires two major transcriptional regulators; the general nitrogen-control protein NtcA―which mediates the response to nitrogen availability―and the differentiation-specific protein HetR [1]. The differentiation of an excessive number of vegetative cells into heterocysts is prevented by diffusible inhibitors produced by prospective heterocysts (proheterocysts) and heterocysts, including morphogens related to the *patS* [2,3] and *hetN* [4,5,6] gene products that act by inhibiting HetR [7,8]. In the mature diazotrophic filament, heterocysts are provided with reduced carbon by the vegetative cells and, in turn, vegetative cells are provided with fixed nitrogen by the heterocysts [8]. Exchanged nutrients appear to include sucrose, glutamate and alanine transferred from vegetative cells to heterocysts [9,10,11], and glutamine and the dipeptide β-aspartyl arginine transferred from heterocysts to vegetative cells [10,12]. The β-aspartyl arginine dipeptide is the product of degradation of cyanophycin (multi-l-arginyl-poly [l-aspartic acid]), a nitrogen reserve material that accumulates at the heterocyst poles. Hence, intercellular molecular transfer is a hallmark of the biology of heterocyst-forming cyanobacteria.

In recent years, three main experimental approaches have enabled a substantial advance in our understanding of intercellular molecular transfer in the heterocyst-forming cyanobacterium *Anabaena* sp. strain PCC 7120 (hereafter *Anabaena*) model. First, the reevaluation of images obtained by transmission electron microscopy (TEM) that have long been available [13] and further electron microscopy studies revealed relevant features of the supracellular structure of the *Anabaena* filament. Second, the use of fluorescent markers led researchers follow intercellular molecular exchange in real time and quantify exchange/transfer parameters [14]. Third, the study of some mutants that were initially characterized by their filament fragmentation phenotype identified proteins involved in intercellular molecular transfer [15,16,17]. In this article, we review these initial findings and further developments that led to the hypothesis that proteinaceous structures termed “septal junctions” mediate intercellular molecular exchange in heterocyst-forming cyanobacteria. We further reevaluate some available intercellular molecular exchange data suggesting that septal junctions are regulated, which is consistent with previously reported findings [9] and recently reported regulation of the septal junctions by gating [18].

## 2. Structure of the Cyanobacterial Filament

The cyanobacteria have a gram-negative type of cell envelope, in which a peptidoglycan (or murein) mesh and an outer membrane lie outside of the cytoplasmic membrane (CM) [19]. In filamentous cyanobacteria, the outer membrane (OM) is continuous along the filament, not entering the septum between contiguous cells (see examples in References [15,20]; reviewed in References [13,19]). This localization of the OM determines the presence of a continuous periplasm, which is the space that lies between the CM and OM and contains the peptidoglycan mesh [13,21]. The peptidoglycan is thicker in cyanobacteria than in well-studied gram-negative bacteria such as the enterobacteria, in which there is only one layer of peptidoglycan, and in *Anabaena* two layers of peptidoglycan appear to surround each cell in the filament [19]. In the septum, the peptidoglycan layers of adjacent cells appear to be frequently joined to each other producing a structure that appears especially dense in TEM analysis of isolated murein sacculi [22,23]. We shall refer to these structures as “septal (peptidoglycan) disks”. Because of this linkage between the peptidoglycans of adjacent cells, murein sacculi corresponding to several cell units can be isolated from filamentous cyanobacteria [23,24,25]. Interestingly, septal disks contain an array of perforations termed “nanopores” (“pores” in an early publication [24]) that are essential for intercellular molecular exchange [22]. Nanopores have a diameter of about 15 to 20 nm [9,22]. Cell wall amidases of the AmiC type responsible for drilling the nanopores in the septal peptidoglycan have been identified [22,26]. This type of amidases splits the septal peptidoglycan for daughter cell separation to complete cell division in unicellular bacteria. With few exceptions, heterocyst-forming cyanobacteria have a gene cluster consisting of *amiC1-amiC2-murI* (genomes checked at https://img.jgi.doe.gov/cgi-bin/m/main.cgi), in which *murI* encodes a glutamate racemase, and many strains also have *amiC3* in a different genomic location. In *Anabaena*, inactivation of any of the three *amiC* genes somehow affects nanopore formation [26,27].

TEM studies of heterocyst-forming cyanobacteria using staining with potassium permanganate showed the presence of thin structures perpendicular to the CM, termed “microplasmodesmata”, that bridge the gap between the adjacent cells in the septa [28,29]. Similar structures were observed and termed “septosomes” in an electron tomography study when potassium permanganate staining was also used [30]. Staining with potassium permanganate suggests these structures are of a proteinaceous nature [30]. Electron tomography studies using staining that highlights the cell wall revealed clear areas, similar to holes or “channels”, traversing the septal peptidoglycan [30,31]. Because of their size, about 14 nm in diameter, these channels could correspond to the nanopores described above and could accommodate the septosomes observed with potassium permanganate, which are about 5.5 nm in diameter [30].

The term microplasmodesmata was also used to denote structures seen as pits (in the exoplasmic fracture face) and protrusions (in the protoplasmic fracture face) in freeze-fracture electron microscopy studies of the CM of the septal regions [32]. Such structures have a mean diameter of about 7–8 nm, and are generally <20 nm in diameter [32]. Hence, they can correspond to the structures that traverse the nanopores. The number of microplasmodesmata or nanopores per septum varies depending on the heterocyst-forming cyanobacterium considered, and probably also on growth conditions. In septa between vegetative cells, 100 to 250 microplasmodesmata were reported for cyanobacteria such as *Anabaena variabilis* and *Anabaena cylindrica* [33], about 155 nanopores per septal disk for *Nostoc punctiforme* [22], and about 75 [9] or 50 [23] nanopores per septal disk in *Anabaena*. The septa between vegetative cells and heterocysts are smaller than those between vegetative cells and, accordingly, have a lower number of microplasmodesmata (about 20% as compared to those between vegetative cells in *A. cylindrica* [32]). The length of the septosomes or the channels has been described in a few cases. Wilk et al. reported a length of about 26–27 nm for the septosomes [30] and Omairi-Nasser et al. reported a length of about 12 nm for the channels between vegetative cells and 21 nm for those between vegetative cells and heterocysts [31], all referring to *Anabaena*.

**Figure 2:**
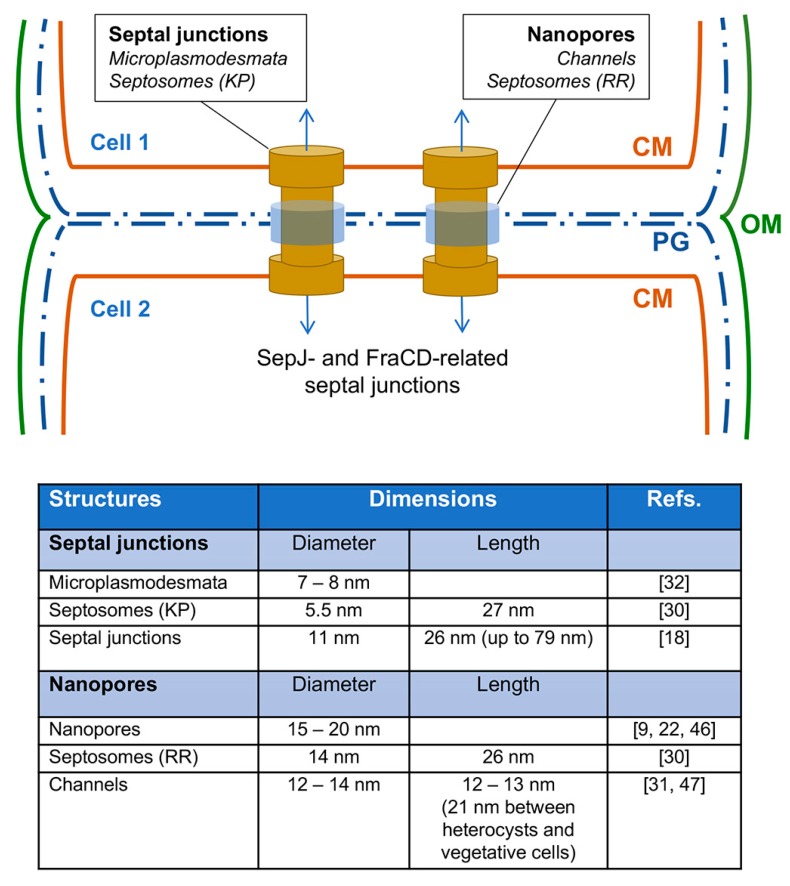
Septal junctions and nanopores in heterocyst-forming cyanobacteria. (**Top**) Schematic of the region between two adjacent vegetative cells. Proteinaceous septal junction complexes and the nanopores through which such complexes traverse the septal peptidoglycan are shown (drawing not to scale). The SepJ- and FraCD-related septal junctions likely contain additional proteins. Whether the nanopores are simply holes in the peptidoglycan or have a special structure is unknown. CM, cytoplasmic membrane; OM, outer membrane; PG, peptidoglycan. (**Bottom**) Dimensions of septal junction-related structures (for simplicity, mean values are presented). The structures are named as in their original description (see References). Septosomes reported by Wilk et al. (2011) [30] were visualized differently by staining with potassium permanganate (KP; positive staining) or ruthenium red (RR; negative staining) and correspond, respectively, to septal junctions or nanopores.

Overall, electron microscopy studies have shown the presence of structures joining the cells in the cyanobacterial filaments. Rather than being lipidic membranes, these structures appear to be made of protein [30,32]. We now term these structures “septal junctions” [34,35,36]. To provide communication between the adjacent cells in the filament, the septal junctions have to traverse the septal peptidoglycan (“cell wall”), and the nanopores observed in septal peptidoglycan disks, or the “channels” observed in some electron tomography studies, are the likely perforations that accommodate the septal junctions (see Figure 2, which includes a summary of the dimensions of septal junction-related structures). A major recent advance in this field has been the visualization of septal junctions with electron cryotomography [18]. The septal junctions observed are described as consisting of protein membrane complexes including cap and plug modules and a CM-anchored tube crossing the intercellular space between the adjacent cells. Interestingly, the tube is 11-nm wide (lumen, 7 nm), which is consistent with the size of the septal junctions observed previously with other electron microscopy techniques and described above. However, the length of the tube varied between 26 and 79 nm depending on its localization, i.e., centered in the septum or closer to its edge, respectively [18].

## 3. Intercellular Molecular Transfer of Fluorescent Markers

The use of fluorescent markers led researchers observe and quantify intercellular molecular transfer in the cyanobacterial filaments in real time [14]. The markers that have been used are calcein (622.5 Da, four ionizable groups only partially negative at pH 7), 5-carboxyflurescein (5-CF; 376.3 Da, one negative charge), and esculin (a coumarin glucoside analog of sucrose, 340.3 Da, mostly neutral at pH 7) [9,14,37]. Calcein and 5-CF are loaded as hydrophobic esterified precursors into the cells, where they are hydrolyzed producing highly hydrophilic and fluorescent compounds that are retained in the cytoplasm [14,38], whereas esculin is taken up by glucoside transporters [9,39]. Once inside the cells, the markers can be excited with monochromatic light for visualization and, by applying light of high intensity can be destroyed, resulting in loss of fluorescence. Bleaching is followed by recovery of fluorescence in the bleached cell, which enables fluorescence recovery after photobleaching (FRAP) analysis that can be quantified to calculate molecular exchange coefficients [14] or, more simply, recovery rate constants [38,39]. Fluorescence recovered in the bleached cell corresponds to fluorescence lost from the adjacent cells [14], indicating movement of the marker and, because movement is always down the concentration gradient [9,14,38], suggesting that it takes place by diffusion. A study of the temperature dependence of FRAP analysis in *Anabaena* has shown that intercellular molecular transfer of the three tested markers (calcein, 5-CF, esculin) is directly proportional to the absolute temperature, indicating that the process indeed takes place by simple diffusion [40]. Additionally, Kang et al. calculated the activation energy (*E_a_*) for the intercellular transfer of the three markers [41], suggesting that dissociation of water molecules from the markers may be involved in the diffusion process.

Intercellular transfer of fluorescent markers is generally faster between vegetative cells of filaments incubated in the absence of combined nitrogen for between 16 hours and 3 days than between vegetative cells from filaments grown in the presence of combined nitrogen [9,14,37]. However, transfer from vegetative cells to heterocysts is slower than between vegetative cells of the same filaments [9,14,39].

## 4. Septal Proteins and Septal Junctions

Filaments of cyanobacteria such as *Anabaena* can be hundreds of cells long, and mutants that make only short filaments (the filament fragmentation phenotype) have been identified among mutants showing a Fox^–^ phenotype (inability to grow fixing N_2_ under oxic conditions) [42,43]. The study of some of these mutants resulted in the identification of integral membrane proteins SepJ [16] (also known as FraG [17]), FraC and FraD [15,44], which are located at the cell poles in the intercellular septa [16,44] (as an example, see the subcellular localization of a SepJ-GFP fusion in Figure 1B). These proteins have been discussed in detail elsewhere [8] and will be described here only briefly.

The SepJ protein (product of ORF *alr2338* from the *Anabaena* genome) has four well-conserved domains: (i) an N-terminal sequence of about 26 amino acids; (ii) a coiled-coil domain (amino acid residues 28 to 207 of the *Anabaena* protein) with two strongly predicted coiled-coil motifs; (iii) a linker domain rich in Pro, Ser and Thr (residues 208–411); and (iv) an integral membrane or permease domain (residues 412–751). The C-terminus of the protein is clearly cytoplasmic [16,45], whereas the N-terminal section (comprising domains i to iii) has been considered to be extracellular [16,45,46], although this interpretation has been challenged [47]. Recent work indicates that SepJ, and in particular its coiled-coil domain, is a peptidoglycan-binding protein [48], strongly suggesting a periplasmic localization of this domain of SepJ. Interestingly, the conserved N-terminal sequence (domain i) may be removed from the mature SepJ protein [48]. Based on bacterial adenylate cyclase two hybrid (BACTH) analysis and on studies of proteins solubilized from cellular membranes by mild treatments, SepJ has been shown to form multimers [45,48].

FraC and FraD are encoded in an operon with the structure *fraC-fraD-fraE* (ORFs *alr2392-alr2393-alr2394* in the *Anabaena* genome) which is strongly conserved in heterocyst-forming cyanobacteria [44]. The three proteins encoded in this operon are integral membrane proteins, and FraC and FraD are septal proteins [44]. (Unfortunately, no information on the subcellular localization of FraE is available.) FraD has an N-terminal domain consisting of five transmembrane segments (residues 1–172) and a C-terminal extra-membrane peptide (residues 173–343) located in the periplasm [38]. Because of conservation of gene clustering, and because their mutants show identical phenoptypes, FraC and FraD are thought to work together.

The Δ*fraC* and Δ*fraD* mutants show impaired localization of SepJ at the cell poles [44], which might suggest a relationship between the three proteins. However, no further evidence for interaction between FraCD and SepJ has been found (e.g., BACTH analysis negative; Flores, unpublished). Furthermore, whereas the *sepJ* mutants are arrested in heterocyst differentiation [16,17], the *fraC*, *fraD* and double *fraC fraD* mutants form heterocysts that are active, although showing low nitrogenase activity [38,44]. Therefore, a direct relationship between SepJ and FraCD remains to be established.

The mechanism of filament fragmentation of the *sepJ*, *fraC* or *fraD* mutants appears to be related to the action of the AmiC amidases that drill the nanopores in the septal peptidoglycan [26]. Thus, double *sepJ amiC1* or triple *fraC fraD amiC1* mutants show a significantly decreased filament fragmentation phenotype when compared to the corresponding *sepJ* or *fraC fraD* mutants, indicating that AmiC1 is largely responsible for the fragmentation of the filaments in the mutants of the septal proteins [26]. It is possible that in the absence of the septal proteins, AmiC1 activity is deregulated resulting in the splitting of septal peptidoglycan and, in turn, in filament fragmentation.

Consistent with a possible functional relationship between the septal proteins and the AmiC amidases that drill the nanopores, the Δ*sepJ* and Δ*fraC* Δ*fraD* mutants are impaired in nanopore formation [9]. The Δ*sepJ* and Δ*fraC* Δ*fraD* mutants show, respectively, about 14% and 12% of the nanopores found in wild-type *Anabaena* [9]. Additionally, an *Anabaena* strain overexpressing SepJ produces an increased number of nanopores [23]. These observations suggest that both SepJ and FraCD are involved in nanopore formation. On the other hand, the mutants of the septal proteins are characteristically impaired in the intercellular transfer of fluorescent markers [9,14,44], with the triple Δ*sepJ* Δ*fraC* Δ*fraD* mutant showing about 28–29% of 5-CF and calcein transfer and about 50% of esculin transfer as compared to the wild type [9]. Overall, these observations suggest that SepJ and FraCD are needed to form septal junctions, which are involved in the formation of nanopores and in the intercellular transfer of fluorescent markers.

## 5. Is There More than One Type of Septal Junction?

Whereas the Δ*fraC* and Δ*fraD* mutants are similarly impaired in the intercellular transfer of calcein and 5-CF, the Δ*sepJ* mutant is more affected in the transfer of calcein than of 5-CF [9,38], and a double Δ*fraC* Δ*fraD* mutant is more affected than the Δ*sepJ* mutant in the transfer of esculin [9]. Based on the differential effects that the inactivation of *sepJ*, on one hand, and of *fraC* and *fraD*, on the other, have on the intercellular transfer of fluorescent markers, we have suggested the existence of at least two types of septal junctions, those related to SepJ and those related to FraCD [9,38]. As mentioned earlier, calcein is larger than 5-CF or esculin, and therefore SepJ-related septal junctions might allow diffusion of somewhat larger molecules than FraCD-related septal junctions. The recently visualized septal junctions lack their cap and plug modules in Δ*fraC* and Δ*fraD* mutants, corroborating an important role of FraC and FraD in the formation of those septal junctions [18].

In electron microscopy tomographic studies of *Anabaena*, the Δ*sepJ* mutant has been observed to still contain “septosomes” [30] or “channels” [47]. Wilk et al. reported that the distance between the adjacent vegetative cells in the septa of this mutant was reduced as compared to that in the wild type. If the septal junctions determine the distance between the cells, the septal junctions remaining in the Δ*sepJ* mutant may be shorter than those present in the wild type [30]. In contrast, Omairi-Nasser et al. reported that the “channels” remaining in the Δ*sepJ* mutant are of a similar length as those in the wild type [47]. This observation can be readily understood because whereas different septal junctions could have different lengths, the “channels” are perforations in the septal peptidoglycan, and channel length should therefore correspond to septal peptidoglycan thickness. A reduced distance between the CM of adjacent cells has also been reported for the triple Δ*sepJ* Δ*fraC* Δ*fraD* mutant as compared to the wild type [9]. This mutant still forms nanopores (7% of those in the wild type) and, as described above, shows reduced but significant intercellular transfer of the fluorescent tracers [9]. It is therefore possible that septal junctions made of other components are formed in the triple Δ*sepJ* Δ*fraC* Δ*fraD* mutant [9]. If different types of septal junctions do exist, it is intriguing why inactivation of either *sepJ* or *fraC* and *fraD* results in about 10% the number of nanopores observed in *Anabaena*. Perhaps regulation of AmiC1 to drill nanopores instead of splitting the septal peptidoglycan requires the presence of both SepJ and FraCD.

An alternative hypothesis to that of the presence of multiple types of septal junctions is that SepJ provides a dock for the construction of septal junctions but is not itself a component of any type of junction [47]. However, this hypothesis does not explain the differential effects of the inactivation of different septal proteins on the intercellular transfer of fluorescent markers described in the previous paragraphs. Additionally, the integral membrane (permease) domain of SepJ from a non-heterocyst forming cyanobacterium (*Trichodesmium erythraeum*) provides SepJ with its structural role (formation of long filaments) but does not provide the diazotrophic function contributed by the permease domain of *Anabaena* [37]. We have also recently reported that changes in some particular amino acid residues of the SepJ permease domain alter intercellular transfer of calcein without affecting diazotrophic function, indicating subtle differences in intercellular molecular transfer [49]. These observations point to a role of SepJ as a component of some septal junctions.

## 6. Junctions in the Vegetative Cell-Heterocyst Septa

The information summarized above refers mainly to septal junctions between vegetative cells. The septa between vegetative cells and heterocysts are smaller than those between vegetative cells as a result of the constriction of the heterocyst at its poles forming “heterocyst necks” (see for example, Figure 1 in Reference [36]). This results in the presence of a lower number of putative septal junctions (“microplasmodesmata” in Reference [32]) in these septa. On the other hand, as mentioned above, the “channels” described by Omairi-Nasser et al. appear to be longer in vegetative cell-heterocyst septa than in septa between vegetative cells [31], perhaps reflecting a thicker peptidoglycan in the former than in the latter. Even longer “channels” have been observed in the Δ*fraC* Δ*fraD* mutant [47], consistent with the detection of long positively-stained structures observed in the form of thin strings in the Δ*fraC* mutant [38]. These results suggest that either other components of the FraCD-related septal junctions or other types of septal junctions take an altered form in the *fraC* and/or *fraD* mutants.

An intriguing aspect linked to the differentiation of the heterocyst neck is that a SepJ-GFP fusion protein normally seen as one spot of fluorescence in the septa between vegetative cells, splits into two spots in vegetative cell-heterocyst septa [16,36], even spreading towards the interior of the heterocyst neck [47]. It is possible that the SepJ-GFP protein present in the septa between vegetative cells re-localizes upon heterocyst differentiation, but a topological problem arises because immunolabeling of the coiled-coil domain of SepJ passes from the periplasmic space to the interior of the heterocyst neck, where the cyanophycin plug is located [47]. Because SepJ has a very hydrophobic permease domain, it should be anchored to a lipidic membrane. Although the cyanophycin plug has generally been considered not to be surrounded by a membrane, an electron-dense line is normally observed around the plug (discussed in Reference [36]; see also Figure S3 in Reference [50]), and at least one report suggesting the presence of a membrane is available [51].

The transfer of fluorescent markers from vegetative cells to heterocysts is slower than between vegetative cells of the same filaments: about 90% slower in the case of calcein [14] and about 60% slower in the case of esculin [9]. This is consistent with the presence of a lower number of putative septal junctions in the vegetative cell-heterocyst septa than between vegetative cells (“microplasmodesmata” in Reference [32]). Additionally, the cyanophycin plugs also seem to slow down transfer of calcein to heterocysts [14], although this effect is not observed for esculin [9]. On the other hand, overexpression of SepJ results in a significant increase of calcein transfer from vegetative cells to heterocysts [23]. Overall, available data suggest the presence of both FraCD- and SepJ-related junctions in the septa between vegetative cells and heterocysts, although some of their properties may differ from those of the junctions present in the septa between vegetative cells.

## 7. Further Mutations Affecting the Septal Junctions

As described above, the *sepJ*, *fraC* and *fraD* mutants are impaired in intercellular molecular transfer studied with fluorescent markers. Additionally, mutations such as those in the *amiC1* mutants that affect the formation of nanopores impair intercellular transfer of calcein and 5-CF [26], and both parameters (nanopore formation and marker transfer) are affected to a similar extent, about 50% of wild-type values [26]. Thus, not only septal junctions are involved in formation of nanopores, but formation of a normal number of nanopores by AmiC-type amidases is reciprocally needed to observe the full septal junction activity assessed by intercellular molecular transfer.

Other mutations that impair intercellular molecular transfer and somehow affect the formation of nanopores include those that inactivate genes *alr3353* (encoding a peptidoglycan-binding LytM factor [50]), *sjcF1* (encoding a peptidoglycan-binding protein [46]), and *glsC* (encoding a nucleotide-binding domain of an ABC transporter for glucosides [39]). An *alr3353* mutant, although not completely segregated, shows about 50% of the nanopores and of the transfer of fluorescent markers (calcein, 5-CF) that are observed in the wild type, and the Alr3353 protein has been shown to interact with AmiC1 [50]. Alr3353 may directly activate AmiC1 to drill nanopores that are necessary for intercellular transfer of calcein and 5-CF [50]. Similar to *alr3353*, a *sjcF1* insertional mutation could not be segregated, but the partially segregated mutant shows nanopores with an altered diameter (29 ± 8.2 nm vs. 19 ± 3.5 nm in the wild type) and a decreased calcein transfer (about 33% that in the wild type) [46]. SjcF1 is involved in protein-protein interactions with FraC (but not FraD) and SepJ, and it has been suggested that it links nanopore formation to septal junctions involving FraCD and SepJ [46]. A *glsC* inactivation mutant is impaired in the intercellular transfer of calcein and esculin but not of 5-CF, and it makes about 50% of nanopores compared to the wild type [39]. Overall, these results (i) corroborate that the formation of nanopores is required for formation and operation of the septal junctions; and (ii), indicate that nanopore formation, which requires AmiC-type amidases, is influenced by a number of proteins including peptidoglycan-binding proteins and other proteins such as GlsC that may influence nanopore formation indirectly.

Inactivation in *Anabaena* of further components of the ABC glucoside transporter Gls, as well as inactivation of the major facilitator superfamily (MFS) glucoside transporter HepP, impairs intercellular transfer of the fluorescent markers to different extents [39,52]. In contrast to the mutations described in the previous paragraph, these mutations do not affect nanopore formation. Interestingly however, HepP and the membrane components (GlsP, GlsQ) of the ABC transporter Gls have been shown to interact with SepJ in BACTH analysis [39,52], suggesting that interaction with several membrane transporters may influence, and perhaps regulate, molecular transfer through the septal junctions [53].

## 8. Are the Septal Junctions Regulated?

In a study labeling diazotrophic filaments of *Anabaena* with esculin, the presence of some heterocysts that did not get labeled with esculin was observed, in contrast to heterocysts that showed similar labeling as their neighboring vegetative cells [9]. Those heterocysts were defined as non-communicating heterocysts, and their fraction increased as the heterocyst population aged [9]. This observation indicates that a vegetative cell-heterocyst septum can have all its esculin-transferring septal junctions closed and suggests that septal junctions can be regulated. Because the septal junctions appear to provide conduits allowing diffusion between adjacent cells, gating would be a possible mode of regulation.

**Figure 3:**
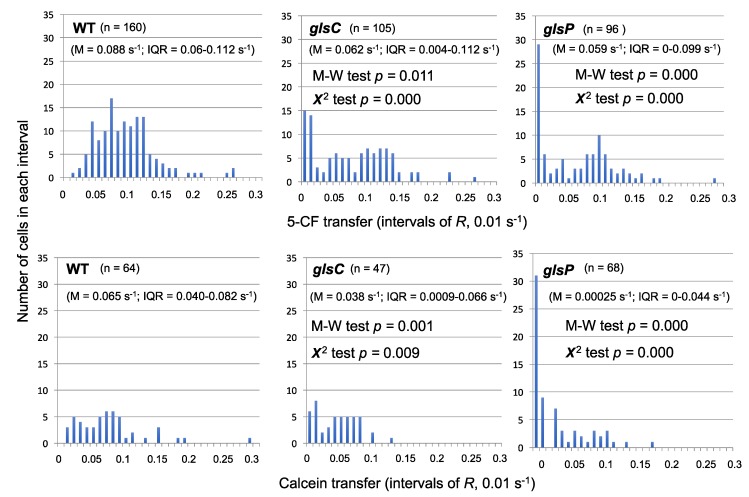
Intercellular transfer of calcein and 5-CF between vegetative cells of filaments from wild-type *Anabaena* and mutants *glsC* and *glsD* grown with combined nitrogen (BG11 medium) (the raw data are the same as those used in Reference [39]). The recovery rate constant, *R*, is organized in intervals of 0.01 s^−1^, and the number of cells in each interval is presented. The number of cells subjected to FRAP analysis (n), the median value of *R* (M) and the interquartile range (IQR) are indicated for each strain and marker, and the difference between each mutant and the wild type for each marker was analyzed by the Mann-Whitney and χ^2^ tests. For the latter, the cells were distributed in groups with *R* < 0.01 or *R >* 0.01 s^−1^.

We have reevaluated some of the fluorescent marker transfer data obtained with some of the mutants described above and observed the presence of a high proportion of non-communicating cells. A clear example was found with 5-CF and calcein transfer in *Anabaena* mutants of the *glsC* and *glsP* genes. Figure 3 shows the distribution of cells with different values of 5-CF (top) or calcein (bottom) transfer. Whereas the wild type exhibited a normal distribution of the data (clearer for 5-CF than for calcein), the mutants presented an especially high number of cells with recovery constant (*R*) values of less than 0.01 s^−1^ (most of them <0.001 s^−1^), which we define as non-communicating cells. We have previously compared the data from the mutants and the wild type with the Student’s *t*-test, concluding that 5-CF and calcein transfer in the mutants was significantly different than in the wild type [39]. However, because the distribution of the data in the mutants is hardly a normal distribution, we applied the Mann-Whitney test, which corroborated that the difference in 5-CF and calcein transfer between each mutant and the wild type was significant (M-W test, *p* < 0.05; Figure 3). Additionally, to assess whether the number of non-communicating cells was significantly different in the mutants and the wild type, we used the χ^2^ test to compare the distribution of cells with *R* < 0.01 s^−1^ and *R* > 0.01 s^−1^, and we obtained a significant difference for both markers and both mutants as compared to the wild type (χ^2^ test, *p* < 0.01; Figure 3). We suggest that inactivation of the *glsC* and *glsP* genes alters the regulation of the septal junctions resulting in an abnormal number of cells with all (or most) of their septal complexes closed. Additionally, as mentioned above, inactivation of *glsC* impairs the formation of a normal number of nanopores [39]. Hence, the ABC transporter Gls (a transporter for glucosides including sucrose [39,52]) appears to be necessary to keep septal junctions open. An appealing hypothesis is that the Gls transporter transmits information on the availability of sucrose to the septal junctions. Further work will be necessary to test this hypothesis.

**Figure 4:**
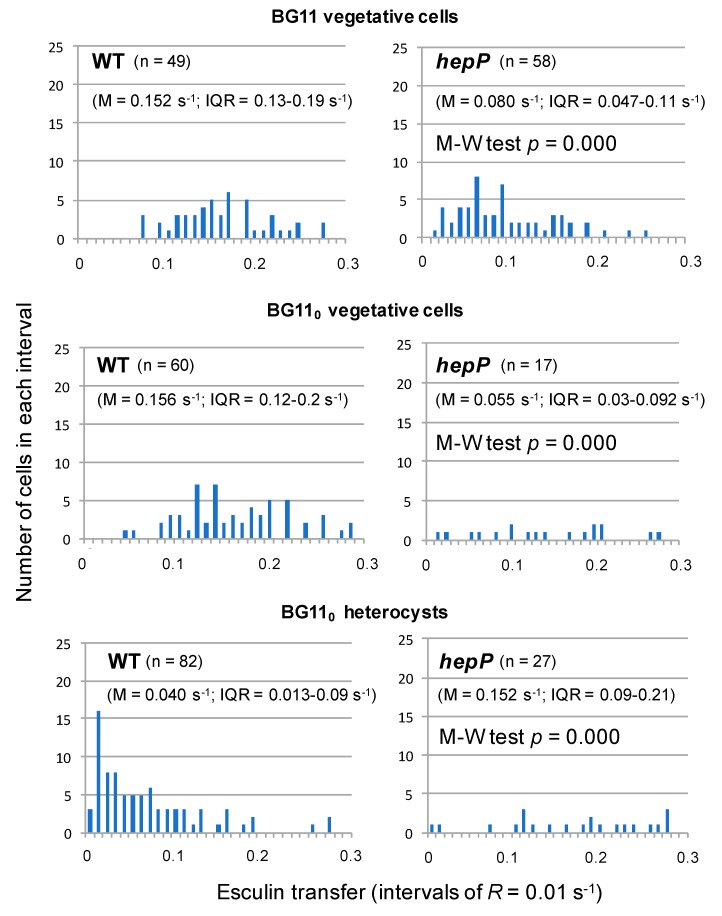
Intercellular transfer of esculin between vegetative cells of filaments from wild-type *Anabaena* and mutant *hepP* grown with combined nitrogen (BG11 medium), or between vegetative cells and from vegetative cells to heterocysts in filaments incubated for 48 h in the absence of combined nitrogen (BG11_0_ medium) (the raw data are the same as those used in Reference [39]). The recovery rate constant, *R*, is organized in intervals of 0.01 s^−1^, and the number of cells in each interval is presented. The number of cells subjected to FRAP analysis (n), the median value of *R* (M) and the interquartile range (IQR) are presented for each type of cell and strain. The difference between the mutant and the wild type was analyzed with the Mann-Whitney test.

Possible regulation involving the MFS glucoside transporter HepP is complex. A *hepP* inactivation mutant was impaired in transfer of esculin and calcein (but not of 5-CF) between vegetative cells of nitrate-grown filaments [39]. Esculin transfer was also determined in diazotrophically-grown filaments of the *hepP* mutant, in which, as compared to the wild type and analyzed with the Student’s *t*-test, transfer was significantly impaired between vegetative cells but stimulated from vegetative cells to heterocysts [39]. Analysis with the Mann-Whitney test corroborated these differences were significant (Figure 4). Non-communicating cells were however not observed to accumulate in the *hepP* mutant. Instead, a relatively high number of low-communicating heterocysts were observed in the wild type (Figure 4). Hence, HepP is necessary for full transfer activity through septal junctions between vegetative cells but appears to restrict transfer from vegetative cells to heterocysts. It will be of interest to investigate whether there are physiological conditions (e.g., conditions determining a high sucrose supply) under which HepP is not restrictive for esculin transfer to heterocysts.

Subjecting *Anabaena* to ionophore-induced or oxidative stress has been found to decrease the number of communicating cells assessed by calcein FRAP analysis [18]. Because this effect can be reversed by incubation of the cells under non-stress conditions and is accompanied by a structural alteration of the septal junctions observed by electron cryotomography, the authors have suggested regulation of the septal junctions by gating [18]. Overall, available information suggests that the septal junctions that connect the cells in the *Anabaena* filament can be closed or opened depending on physiological conditions that may require communicating or isolated cells respectively, and that different membrane proteins (transporters) influence this regulation.

## 9. Physiological Substrates of the Septal Junctions

Calcein and 5-CF are artificial markers that mimic hardly any physiological compound, whereas esculin is a sucrose analog [9]. It is therefore of interest that intercellular transfer of esculin is affected in a Δ*fraC* Δ*fraD* mutant more than in a Δ*sepJ* mutant [9], suggesting that the intercellular transfer of sucrose in *Anabaena* takes place more through FraCD-related than through SepJ-related septal junctions. On the other hand, genetic analysis has shown that HetN-dependent [54] and PatS-dependent [55] signaling is altered in *sepJ* mutants, suggesting that SepJ-related septal junctions are involved in the intercellular transfer of the HetN- and PatS-related morphogens. Additionally, heterocyst patterning is altered in an *Anabaena* strain that overexpresses SepJ, and this effect is alleviated in *patS* or *hetN* mutants, strongly supporting a role of SepJ in the intercellular transfer of the morphogens [23]. A role of SepJ-related septal junctions in the transfer of regulators of heterocyst differentiation and a major role of FraCD-related septal junctions in the transfer of metabolites help us to understand the phenotypes of Δ*sepJ* and Δ*fraC* Δ*fraD* mutants. Whereas the former is blocked in heterocyst differentiation at an early stage [16], which could result from the accumulation of inhibitors in the differentiating cells, the latter form heterocysts [44]. Nonetheless, the heterocysts formed in Δ*fraC*, Δ*fraD* or double Δ*fraC* Δ*fraD* mutants show low nitrogenase activity [38,44], which could result from a shortage of sucrose received from vegetative cells.

## 10. Concluding Remarks

Septal junctions are essential for the behavior as multicellular organisms of heterocyst-forming cyanobacteria, in which cells exchange regulators and nutrients to grow under diazotrophic conditions. As we have previously discussed [9,40], the cyanobacterial septal junctions resemble the metazoan gap junctions, and more precisely their constituent connexons, which are protein complexes that allow the diffusion of small compounds and ions between cells and can be regulated by gating [56]. Septal and gap junctions represent therefore a wonderful example of convergent evolution, in which different proteins and structures perform the same function (cell-cell communication) in extremely distant organisms.
